# The influence of walking performed immediately before meals with moderate fat content on postprandial lipemia

**DOI:** 10.1186/1476-511X-4-24

**Published:** 2005-10-06

**Authors:** Martina Pfeiffer, Tanja Ludwig, Caspar Wenk, Paolo C Colombani

**Affiliations:** 1 INW Nutrition Biology, Department of Agriculture and Food Science, Swiss Federal Institute of Technology Zurich, Universitätsstrasse 2, 8092 Zurich, Switzerland

## Abstract

**Background:**

Postprandial lipemia is an independent risk factor for coronary heart disease. Single bouts of moderate exercise may lower this risk, but the minimum duration of moderate intensity exercise that still lowers postprandial lipemia is not known. We, therefore, performed a dose-response study with a normal, daily life setting, to identify the minimum duration of moderate intensity walking that lowers postprandial lipemia in sedentary, healthy young men.

**Methods:**

Sixteen men performed three activity trials (30, 60, or 90 min of treadmill walking at 50% of their individual VO_2_max) and a control trial with no physical activity in a repeated measures crossover design. The subjects walked immediately before ingestion of the first of two mixed meals, which were served 3 h apart. The meals had a moderate fat content (0.5 g per kg body mass and 33% of total energy per meal) and a macronutrient composition corresponding to current recommendations. Each meal provided one third of the subject's estimated daily energy requirement. Venous blood samples were taken in the fasted state, and then hourly for 6 h after the first meal to assess the postprandial phase. Postprandial lipemia (the incremental area under the curve (dAUC) of triacylglycerol) was compared with a mixed model analysis and Tukey's adjustment.

**Results:**

Postprandial lipemia (dAUC of triacylglycerol) was, compared to the control trial, +2% (P = 1.00), -14% (P = 0.24), and -15% (P = 0.23) in the 30, 60, and 90 min walking trials, respectively.

**Conclusion:**

Moderate intensity walking of 60 and 90 min duration slightly, but insignificantly, reduced postprandial lipemia after two mixed meals with moderate fat content in sedentary, healthy young men, compared to inactivity. Therefore, it should be reconsidered if the acute exercise-induced reduction in postprandial lipemia usually observed in studies using high fat meals is of importance in a real, daily life setting.

## Background

Postprandial lipemia describes the blood triacylglycerol (TAG) content after meal intake, and is an independent risk factor for atherosclerosis and coronary heart disease [1-4]. The latter is the leading cause of death in industrialized countries and is rapidly becoming a primary cause of death worldwide [5]. Interventions that have the potential to attenuate the postprandial lipemic response are, therefore, valuable tools for lowering the risk of cardiovascular diseases.

Endurance exercise is known to positively influence postprandial lipemia [6], and endurance athletes show a lower postprandial lipemic response than sedentary people [7]. However, it seems to be the acute response to single exercise bouts rather than the improved endurance capacity per se that has the favourable effect [8], because postprandial lipemia increases rapidly with detraining [9,10]. Furthermore, the intensity at which endurance exercise is usually performed is rather high, which makes endurance exercise not a very attractive option for reducing the cardiovascular risk for sedentary people.

A single exercise bout with moderate intensity only was also shown to improve postprandial lipemia when performed immediately before meal ingestion, but usually the exercise duration used was one hour or longer [11-13]. Since about 60% of the world's population already does not meet the minimum recommendation of daily physical exercise [14], which is 30 min of moderate intensity activity on most, preferably all, days of the week [15], it is unlikely that sedentary people would engage in exercise of long duration. Therefore, it seems important to determine the influence of moderate exercise with shorter duration on postprandial lipemia, but few studies have investigated this exercise mode. We are aware of two studies in which the exercise bouts were performed just before ingestion of a meal and corresponded to an intensity and duration required for health-maintaining physical activity, e.g. moderate intensity exercise with a duration of less than one hour [16,17]. Whereas Murphy et al. [16] found a significant influence of 30 min of brisk walking (60% of maximal oxygen uptake, VO_2_max) on postprandial lipemia in overweight or obese and older subjects, Petridou et al. [17] found a non-significantly lowered TAG response of 17% after 45 min of cycling at 62% of predicted maximal heart rate in sedentary but otherwise healthy young men.

In postprandial lipemia studies, a high fat meal (e.g. 1 g fat per kg body mass, BM) is usually provided to the subjects [18] to induce a strong increase of postprandial lipemia [19]. However, such high fat meals do not represent normal meals either in absolute or relative fat content, and do not correspond to the current recommendations about macronutrient composition [20]. As even 15–30 g of fat already elevate TAG significantly [18], high fat meals are not a precondition to study postprandial lipemia. In order to obtain information on postprandial lipemia that is relevant to real life, it seems more reasonable to choose normal mixed meals with a moderate fat content, as has been done recently in two studies investigating the influence of exercise on postprandial lipemia [17,21]. Additionally, free-living individuals consume sequential meals during the course of the day, but we know of only one study [16] where more than one meal was provided to the subjects. Since second meal effects of postprandial lipemic responses to mixed meals have been reported [22], the results of the studies with just one test meal do not accurately reflect real life [23].

The aim of the present study was, therefore, to determine the minimum duration of walking with a moderate intensity that would significantly lower postprandial lipemia in sedentary, healthy young men in a normal, daily life setting. The subjects performed three activity trials (30, 60, or 90 min of treadmill walking at 50% of their individual VO_2_max), and a control trial with no physical activity. Immediately after exercising they ingested the first of two mixed meals, which were served 3 h apart. The meals had a moderate fat content (0.5 g per kg BM and 33% of total energy per meal), a macronutrient composition corresponding to current recommendations [20], and each one provided one third of the subject's estimated daily energy requirement.

## Results

### Exercise sessions

Mean relative oxygen uptake with the 30, 60, and 90 min walking sessions was 49.8 (0.2)%, 50.1 (0.1)%, and 50.0 (0.2)% of VO_2_max, respectively. Data comparing mean metabolic responses between the three exercise sessions are presented in Table 1.

**Table 1:** Metabolic responses during 30, 60, and 90 min treadmill walking in the activity trials

	30 min		60 min		90 min	
	
	Mean	SEM	Mean	SEM	Mean	SEM
Energy expenditure [kJ per exercise session]	879^a^	46	1799^b^	83	2630^c^	126
Energy expenditure [kJ·min^-1^]	29	2	30	1	29	1
Respiratory exchange ratio	0.95^a^	0.01	0.92^b^	0.01	0.91^b^	0.01
Fat oxidation [g per exercise session]	4^a^	1	12^b^	2	21^c^	2
Fat oxidation [g·min^-1^]	0.12^a^	0.03	0.20^ab^	0.03	0.23^b^	0.02
Carbohydrate oxidation [g per exercise session]	44^a^	3	80^b^	6	110^c^	6
Carbohydrate oxidation [g·min^-1^]	1.46^a^	0.11	1.33^ab^	0.10	1.22^b^	0.06
Heart rate [min^-1^]	122	4	125	4	127	3
Heart rate [% HRmax]	64	2	65	2	66	1
Rating of perceived exertion*	11.0^a^	0.4	11.5^ab^	0.4	11.8^b^	0.4

SEM, standard error of the mean; HRmax, maximum heart rate.

*Borg rating of relative perceived exertion, 6–20 scale.

Significant difference between means not sharing a common letter (P < 0.05; n = 15 for respiratory data in 30 and 60 min walking trials; n = 16 for other data).

### Blood chemistry

**Figure 1:**
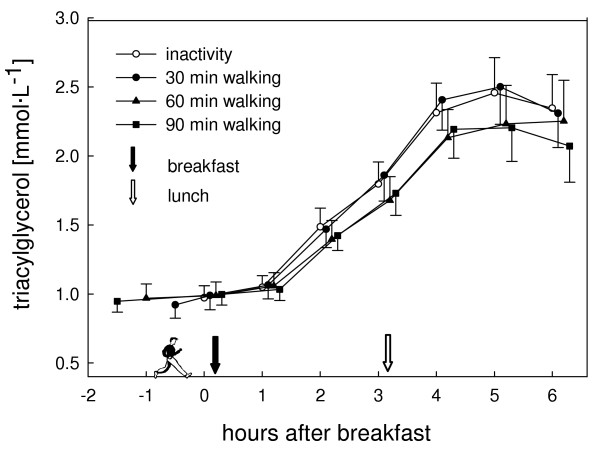
Triacylglycerol concentration for the inactivity and the activity (30, 60, or 90 min treadmill walking) trials. For better readability the symbols of the different trials at the same time points are slightly staggered. Values are means (standard error of the mean). Overall mixed model analysis with repeated measurements, effect of trial: P = 0.06.

**Figure 2:**
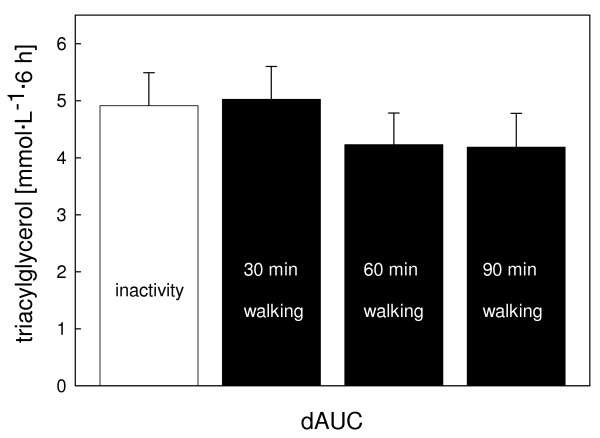
Incremental area under the curve (dAUC) for postprandial triacylglycerol (mean, standard error of the mean) for the inactivity and the activity (30, 60, or 90 min treadmill walking) trials. Overall mixed model analysis, effect of trial: P = 0.07.

The postprandial TAG profile did not differ statistically between the trials (P = 0.06; Fig. 1). Highest values were observed 5 h after breakfast and were 2.63 (0.28) mmol· L^-1^, 2.65 (0.26) mmol· L^-1^, 2.47 (0.28) mmol· L^-1^, and 2.39 (0.26) mmol· L^-1 ^with the control, 30 min, 60 min, and 90 min walking trials, respectively, whereas mean postprandial TAG values (area under the curve (AUC) divided by six) were 1.78 (0.17) mmol· L^-1^, 1.82 (0.17) mmol· L^-1^, 1.71 (0.18) mmol· L^-1^, and 1.71 (0.16) mmol· L^-1^, respectively. Compared to the control trial the increase in AUC (dAUC) of TAG was +2% (P = 1.00), -14% (P = 0.24), and -15% (P = 0.23) for the 30, 60, and 90 min walking trials, respectively (Fig. 2). To detect a statistically significant difference in dAUC of TAG between the inactivity and the 60 or 90 min walking trials a sample size of 279 and 182 subjects, respectively, would have been required (Power = 0.80).

**Figure 3:**
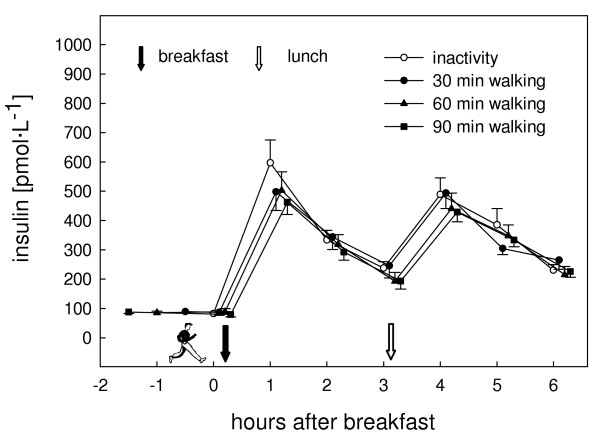
Insulin concentration for the inactivity and the activity (30, 60, or 90 min treadmill walking) trials. For better readability the symbols of the different trials at the same time points are slightly staggered. Values are means (standard error of the mean). Overall mixed analysis with repeated measurements, effect of trial: P = 0.16.

**Figure 4:**
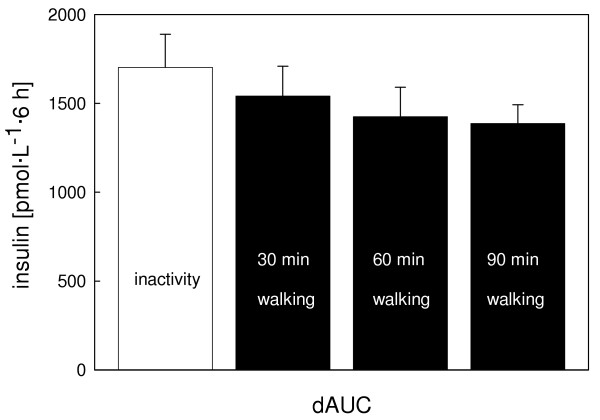
Incremental area under the curve (dAUC) for postprandial insulin (mean, standard error of the mean) for the inactivity and the activity (30, 60, or 90 min treadmill walking) trials. Overall mixed model analysis, effect of trial: P = 0.25.

**Figure 5:**
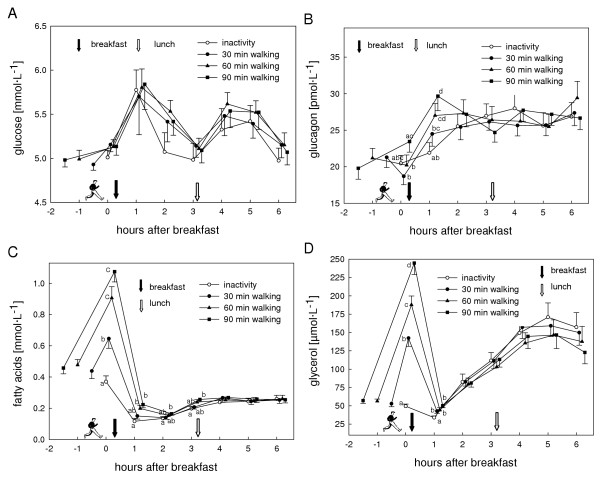
Concentrations of glucose (A), glucagon (B), fatty acids (C), and glycerol (D) for the inactivity and the activity (30, 60, or 90 min treadmill walking) trials. For better readability the symbols of the different trials at the same time points are slightly staggered. Values are means (standard error of the mean). Significant differences between means not sharing a common letter (P < 0.05).

Insulin values were highest one hour after breakfast in all trials. A second peak was observed one hour after lunch (Fig. 3). Compared to the control trial postprandial insulinemia (dAUC) was lowered by 9% (P = 0.79), 16% (P = 0.54), and 19% (P = 0.20) for the 30, 60, and 90 min walking trials, respectively (Fig. 4). There was a significantly lower insulin to glucagon ratio one hour after breakfast in the 90 min exercise trial compared to the control trial (P = 0.01). No difference between the postprandial glucose values of all trials either in the profile over time (P = 0.50) or dAUC (P = 0.95) was observed. Profiles of glucose, glucagon, fatty acids (FA), and glycerol are presented in Figure 5A–D.

## Discussion

In a dose-response study we examined the influence of 30, 60, and 90 min of moderate intensity walking just before meal ingestion to determine the minimum duration necessary to lower postprandial lipemia in sedentary, healthy young men in a normal, daily life setting. Our findings indicate that 60 or 90, but not 30 min of brisk walking induced a modest but statistically insignificant reduction of postprandial lipemia compared to an inactive control.

This is in contrast to most of the available literature, because in most studies a reduction of postprandial lipemia after a moderate intensity exercise bout was found [11-13,16]. The main difference between those studies and the current study was the type or number of test meals. Our test meals had only a moderate fat content (0.5 g per kg BM and 33% of total energy per meal) and were composed of ordinary, commercially available food. Additionally, to simulate the course of a normal day we served two meals (breakfast and lunch), each meal providing one third of the subject's estimated daily energy requirement. In three studies with healthy subjects who exercised immediately before ingestion of a meal, a significant reduction in postprandial lipemia was observed after 60 min walking at 60% of VO_2_max [13], 90 min at 50% of VO_2_max [11], and 90 min at 65% of VO_2_max [12] (postprandial lipemia (dAUC) was lowered by 38%, 49%, and 39%, compared to inactivity, respectively). However, in these studies high fat meals were served (95–101 g, 81–92% of total energy). Since in our study the 60 and 90 min walking sessions did not significantly lower postprandial lipemia, we assume that our test meals with the moderate fat content only and the macronutrient composition according to the current recommendations [20] may have largely contributed to the non-significant response observed.

Besides the amount of fat, the other macronutrients can also influence postprandial lipemia. Carbohydrates in a mixed meal provoke an insulin response, which plays an important role in the regulation of plasma TAG concentration [8], e.g. through down-regulating lipoprotein lipase (LPL) activity in skeletal muscle [24]. This insulin effect is in opposition to the acute effect of exercise (90 min of cycling at 85% of the lactate threshold) that increased muscle LPL activity in the immediate 3 to 4 h after exercise, relative to rest in men [25]. Consequently, it could also be assumed that in our study the suppressing effect of insulin on muscle LPL activity after the meal intake exceeded the stimulating effect of exercise. However, since according to Gill [8] a reduction in postprandial lipemia after moderate intensity exercise would be caused rather through decreased very-low-density lipoprotein (VLDL) production than increased TAG clearance, this is not very likely to have been the case. The moderate exercise sessions elevated FA and glycerol levels significantly, but only for a short time. Nevertheless, it seems relevant and worth mentioning, since it has not always been done in previous studies, that in lipemia studies including exercise, the TAG measurements are corrected for glycerol in order not to overestimate TAG values immediately and up to one hour after exercise.

In one study where three mixed meals with a fat content (47% of total energy) above the recommendation [20] were served, 30 min of brisk walking (60% of VO_2_max) performed before breakfast lowered postprandial lipemia significantly [16]. However, the subjects were overweight or obese and older (34–66 years of age) than in our study (20–30 years). Postprandial lipemia seems to behave differently in healthy, untrained adults of different age groups [26]. Therefore, the influence of an exercise bout on postprandial lipemia might also be influenced by the age of the subjects, and explain the differing findings of that study and ours.

Most similar to our study in terms of design as well as investigated population group was the study conducted by Petridou et al. [17]. In that study, subjects cycled for 45 min at moderate intensity (62% of predicted maximal heart rate) just before ingestion of a meal with a fat content of 0.65 g per kg BM and 35% of total energy. They reported similar results to those in our study, finding also only a modest (-17%) and statistically insignificant effect of the exercise bout on TAG dAUC.

Although postprandial lipemia was not lowered significantly with the exercise session used in this study setting, health benefits due to the exercise bout are nevertheless expected via improved insulin sensitivity, increased energy expenditure, and fat oxidation. Gill et al. [27] stated that in non-diabetic subjects, the insulin response to dynamic metabolic stress is likely to provide the best indirect measure of insulin sensitivity. In this study, postprandial insulinemia tended to decrease the longer the exercise session lasted, with no difference in glycemia, indicating that tissue sensitivity to insulin might have increased [28]. Furthermore, physical activity accumulating an energy expenditure of 4200 kJ per week is associated with a 30% reduction of all-cause mortality rates [29]. Our subjects could reach this weekly amount of energy expenditure by performing, for example, five walking sessions of 30 min, one session of 60 min plus three of 30 min, or one session of 60 min plus one of 90 min. Fat oxidation in these three situations would be 18 g, 23 g, and 32 g per week, respectively, indicating that greater fat oxidation can be obtained when performing fewer but longer activity sessions. Interestingly, five sessions of 30 min equal the current minimum recommendation of physical activity [15]. None of our subjects had difficulties in accomplishing the exercise bouts and, according to the rating of the Borg scale, they perceived the exercise intensity as light. Thus, this kind of physical activity is absolutely suitable to include in daily living.

We conclude that 60 and 90, but not 30 min of moderate intensity walking slightly reduced postprandial lipemia after two mixed meals with moderate fat content in sedentary, healthy young men compared to an inactive control. However, the reduction was not statistically significant. It seems, therefore, that a single exercise session of moderate intensity and with a duration of 90 min or less is insufficient to significantly reduce postprandial lipemia in these subjects in a normal daily life setting, i.e. when normal mixed meals with a moderate fat content are served. However, health benefits can nevertheless be achieved at these exercise intensities through other mechanisms.

## Methods

The study was approved by the Ethical Committee of the Swiss Federal Institute of Technology Zurich and was carried out according to a repeated measures cross over design. All participants of the study gave written informed consent.

### Subjects

Sixteen healthy, normal-weighed, sedentary men participated in the study (Table 2). They were non-smokers and all normotriacylglycerolemic and euglycemic according to the classification of the NCEP [30] and the WHO [31], respectively. One subject had borderline high values of total cholesterol and low-density lipoprotein (LDL) cholesterol while the others were in the normal range (Table 3).

**Table 2:** Characteristics of the male subjects (n = 16)

Characteristic	Mean	SEM
Age [y]	24.8	0.8
Height [m]	1.81	0.02
Body mass [kg]	68.9	1.6
Body mass index [kg· m^-2^]	21.1	0.5
VO_2_max [mL· min^-1^· kg^-1 ^body mass]	41.2	0.8
Maximum heart rate [min^-1^]	192	2

SEM, standard error of the mean; VO_2_max, maximal oxygen uptake.

**Table 3:** Fasting biochemical variables of the subjects (n = 16)

Plasma parameter	Mean	SEM
Triacylglycerol [mmol· L^-1^]	1.03	0.09
Total cholesterol [mmol· L^-1^]	3.99	0.19
HDL cholesterol [mmol· L^-1^]	1.27	0.06
LDL cholesterol [mmol· L^-1^]	2.26	0.15
Glucose [mmol· L^-1^]	5.01	0.08
Insulin [pmol· L^-1^]	91.3	5.0
Glucagon [pmol· L^-1^]	20.2	1.2
Glycerol [μmol· L^-1^]	55.4	3.1
Fatty acids [mmol· L^-1^]	0.43	0.05

SEM, standard error of the mean; HDL, high-density lipoprotein; LDL, low-density lipoprotein.

### Preliminary tests

Each subject performed two preliminary walking tests on a treadmill to determine his fitness capacity. In the first test, which also served to familiarise subjects with the methods of the study, subjects walked twice for approximately 20 min on the treadmill (PULSAR, H-P-COSMOS Sports Medical GmbH, Nussdorf-Traunstein, Germany) at 5 and 6 km· h^-1^, respectively, and with an increasing treadmill inclination of 3% every 3 min. The relationship between work rate and oxygen uptake was established in this test and the results were used to determine the work rate in the activity trials resulting in 50% of VO_2_max.

VO_2_max was determined in the second test. Subjects walked at 6 or 7 km· h^-1^, and the treadmill inclination was increased by 5% every 3 min until exhaustion. The test was designed to be finished after 10 to 12 min. VO_2_max was considered to be valid when at least two of the following three criteria were met: 1) respiratory exchange ratio (RER) >1.1; 2) heart rate within 10 beats per min of the predicted maximum (220 beats per min minus age); 3) rating of perceived exertion ≥19 on the Borg 6–20 scale.

### Main trials

Each subject undertook four randomized trials (one control trial with no activity and three activity trials) at intervals of at least four days. To ensure similar baseline conditions, subjects were only allowed to follow activities of daily life on the two days prior to the test days. On the day preceding the test days, the intake of alcohol and caffeine-containing drinks was limited to at most 1–2 glasses of beer or wine and 2 cups of coffee, or 4 cups of tea, or 1 litre of a caffeine-containing soft drink. Additionally, subjects received a standardized evening meal (spaghetti, tomato sauce, cheese, and apple sauce) to prepare at home, providing 0.2 g fat per kg BM, 2.3 g carbohydrates per kg BM, 0.4 g protein per kg BM, and 53 kJ per kg BM. The subjects arrived at the laboratory by public transport after an overnight fast of at least 12 h. The compliance with the instructions was checked with questionnaires that were filled out by the subjects just after arrival at the laboratory.

A catheter was placed into an antecubital or forearm vein 20 min after arrival and a fasting blood sample (8.7 mL) was taken. Subjects then walked on the treadmill for 30, 60, or 90 min at 50% of their VO_2_max, depending on the respective trial, or rested in the control trial. During walking, respiration and heart rate (Polar Vantage NV, Polar Electro Oy, Kempele, Finland) were measured continuously. The subjects rated their perceived exertion after every 30 min of exercise on the Borg 6–20 scale. The walking session was briefly interrupted after 30 min (in the 60 and 90 min sessions) and after 60 min (in the 90 min session) to flush the catheter with heparin free saline (NaCl 0.9%, B. Braun Medical AG, Emmenbrücke, Switzerland). In the meantime, subjects were allowed to drink water ad libitum. A second fasting blood sample was taken just after the exercise bout. Breakfast was served shortly after, consisting of commercially available cereals, yoghurt, cream, and chocolate drink providing 0.5 g fat per kg BM, 1.6 g carbohydrates per kg BM, 0.4 g protein per kg BM, and 53 kJ per kg BM. Subjects received lunch three hours after breakfast, consisting of commercially available bread, cheese, chocolate cream, cream, and orange juice providing 0.5 g fat per kg BM, 1.6 g carbohydrates per kg BM, 0.5 g protein per kg BM, and 53 kJ per kg BM. Blood samples were taken every hour for six hours after breakfast to assess the postprandial period. The catheter was flushed with heparin free saline every 30 min to keep it patent. Water was available ad libitum during the postprandial period in the first trial, and the ingested amount was replicated in the following trials. The subjects stayed at the laboratory and pursued only seated activities until the end of the test day.

### Indirect calorimetry and energy expenditure calculations

Oxygen uptake and carbon dioxide production during physical activity were determined using a pulmonary gas exchange system (Quark b2, Cosmed, Rome, Italy). Energy expenditure, fat, and carbohydrate oxidation during physical activity were calculated according to the Weir formula [32] and a table of the nonprotein respiratory quotient [33], assuming that the urinary nitrogen excretion was negligible.

### Blood sampling and analyses

Fasting blood samples were analysed for TAG, insulin, glucagon, total cholesterol, LDL cholesterol, high-density lipoprotein (HDL) cholesterol, FA, glycerol, and glucose. Postprandial blood samples were analysed for TAG, insulin, glucagon, FA, glycerol, and glucose. Venous blood was collected into a 7.5 mL EDTA tube for analyses of TAG, insulin, total cholesterol, LDL cholesterol, HDL cholesterol, glycerol, and FA. A protease-inhibitor (Trasylol, Bayer AG, Leverkusen, Germany) was immediately added to an aliquot of the EDTA blood for analysis of glucagon. An additional 1.2 mL EDTA/Fluoride tube was collected for analysis of glucose. The tubes were stored on ice until centrifugation (3700 rpm, 8 °C, 12 min; g = 1800; Omnifuge 2.0 RS, Heraeus Sepatech, Osterode, Germany). After separation of venous samples, aliquots of serum were stored at -80 °C. Analyses were conducted after conclusion of the entire experimental period.

A baseline fasting blood sample from each subject's first experimental day was analyzed by enzymatic colorimetric methods for TAG, total cholesterol (both Hitachi Modular P system, Roche Diagnostics, Basel, Switzerland), HDL (Cobas Integra800 analyzer, Roche Diagnostics, Basel, Switzerland), and LDL (calculated by the Friedewald formula [34]).

All fasting and postprandial blood samples were analysed for TAG, FA, glycerol, and glucose by enzymatic colorimetric methods, using a centrifugal analyzer (Cobas-Mira, Roche, Basel, Switzerland). Quality control sera (Roche Diagnostics, Basel, Switzerland) were used to ensure accuracy and precision. The TAG values were corrected by subtracting the glycerol values. Insulin and glucagon were analysed in duplicates by radioimmunoassay, using half of the prescribed amount of kit reagents (LINCO Research, St. Charles, Missouri, USA). The centrifugation steps (glucagon: 4200 rpm, 4 °C, 30 min and insulin: 4700 rpm, 4 °C, 30 min) were carried out in a conventional laboratory centrifuge (Varifuge RF, Heraeus Sepatech, Hanau, Germany) and radioactivity was measured using a gamma counter (Cobra II, Packard Gamma Counter, Minnesota, USA).

### Data analyses and statistics

The AUC and the dAUC TAG were calculated using the trapezoidal method [35]. The measurement just before meal intake was taken as the base value. Statistical analyses were performed using SAS statistical software (version 8.2 for Windows, SAS institute Inc., Cary, NC, USA). The results of the responses during exercise, AUC, dAUC, and blood measurements at specific time points were compared with a mixed model analysis with Tukey's adjustment. The postprandial responses over time were compared with a mixed model analysis with repeated measurements and Tukey's adjustment. The factors for the analyses were subject, period, and trial. The fasting value before activity was used as an additional factor for the postprandial responses over time. Data are presented as mean and standard error of the mean (SEM). A p-value of less than 0.05 was considered significant.

## Authors' contributions

MP was responsible for designing, planning, and accomplishing the study, the analyses of the results, as well as drafting the manuscript. TL participated in the accomplishment of the study and analyses of the blood samples. CW participated in the design and coordination of the study. PCC participated in designing the study, the analyses of the results, and drafting the manuscript.
